# Spondylodiscitis caused by the *Burkholderia cepacia* complex

**DOI:** 10.1590/0037-8682-0001-2024

**Published:** 2024-03-25

**Authors:** Rulian Christi Souza Rodrigues Candido, Luiz Fernando Monte Borella, Marcelo de Carvalho Ramos, Lucieni Oliveira Conterno, Fabiano Reis

**Affiliations:** 1 Universidade Estadual de Campinas, Departamento de Anestesiologia, Oncologia e Radiologia, Campinas, SP, Brasil.; 2 Universidade Estadual de Campinas, Departamento de Clínica Médica, Campinas, SP, Brasil.

A 68-year-old man with dialytic chronic kidney disease presented with intense and progressive lower back pain extending to the lower limbs over the past four months. He also experienced episodes of intermittent fever.

Laboratory tests showed a white blood cell count of 6.10×10³/µL, a C-reactive protein value of 150 mg/L, and a sedimentation rate of 43 mm/h. Spinal magnetic resonance imaging (MRI) was consistent with spondylodiscitis (Figure 1).

**FIGURE 1:**
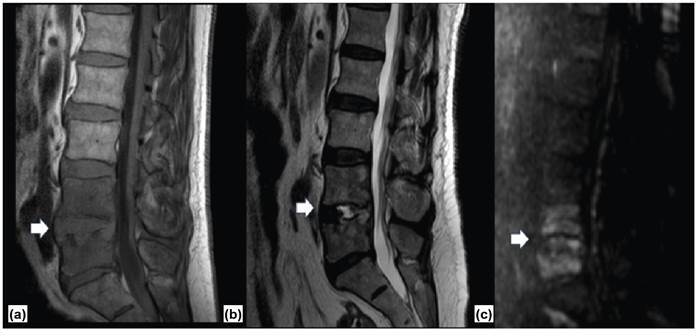
Sagittal images of spine MRI showing an infectious process in the intervertebral disc and vertebral bodies at L4-L5, characterized by hypointensity on T1 weighted images (WI) **(A)**, hyperintensity on T2 WI **(B)** and restricted diffusion on diffusion WI **(C)**. Gadolinium contrast medium injection was not indicated because the patient had a chronic dialytic kidney disease.

Blood cultures were positive for the *Burkholderia cepacia* complex, indicating hematogenous dissemination through the venous catheter. Treatment comprised catheter change, meropenem 0.5 g q24h (dialysis day dose post-dialysis), and levofloxacin 0.5 g q48h. The patient showed improvement in clinical, laboratory, and imaging findings.

 Spondylodiscitis most commonly occurs as a result of hematogenous spread to distant foci; however, it can result from direct spread from a nearby infection or from inoculation during spinal surgery^1^. 

The *Burkholderia cepacia* complex is an under-recognized Gram-negative bacillus that can cause pyogenic spondylodiscitis in patients with chronic kidney disease, malnutrition, substance abuse, HIV infection, diabetes mellitus, long-term steroid use, liver cirrhosis, and malignancy^1^. This report highlights the importance of imaging investigations in patients undergoing hemodialysis with severe low back pain and fever. Owing to the debilitating nature of this disease and multidrug resistance of the bacterium^2^, a precocious diagnosis is paramount. 

Diffusion-weighted images are currently not used for spinal imaging; however, they may be an essential diagnostic tool for spinal infections^3^, particularly in patients with contraindications for gadolinium contrast medium injection.

## References

[1] Subramanian R, Fitzgibbons L (2022). Burkholderia cepacia Complex Lumbar Spondylodiscitis: A Rare Nosocomial Infection. Case Rep Infect Dis.

[2] Hammoud M, Fares Y, Atoui R, Dabboucy B (2019). Burkholderia cepacia as a cause of pyogenic spondylodiscitis in immunocompetent patients: a single-institution case series and literature review. J Spine Surg.

[3] Miyoshi IC, de Toledo AHN, Pereira FV, Villarinho LL, Dalaqua M, de Ávila Duarte J (2023). Infectious Myelitis. Semin Ultrasound CT MR.

